# Self-Assessed Health and Socioeconomic Inequalities in Serbia: Data from 2013 National Health Survey

**DOI:** 10.3389/fphar.2016.00140

**Published:** 2016-05-26

**Authors:** Svetlana Radevic, Sanja Kocic, Mihajlo Jakovljevic

**Affiliations:** ^1^Department of Social Medicine, Faculty of Medical Sciences, University of KragujevacKragujevac, Serbia; ^2^Center for Informatics and Biostatistics, Institute of Public Health KragujevacKragujevac, Serbia; ^3^Health Economics and Pharmacoeconomics, Faculty of Medical Sciences University of KragujevacKragujevac, Serbia

**Keywords:** health surveys, inequalities in health, self-assessed health, socio-economic determinants, Serbia

## Socioeconomic inequalities—impact on health

Health inequities are differences in health status or health determinants between population groups, which are unjust because they reflect an unfair distribution of the underlying social determinants of health (Marmot, 2005; World Health Organization, 2013; Arcaya et al., 2015). This is a global phenomenon, seen in low, middle (Jakovljevic and Getzen, 2016) and high income countries (Ogura and Jakovljevic, 2014).

The largest contribution to inequalities in health is attributable to socio-economic determinants of health, or the societal conditions in which people are born, grow up, live, work, and age, which in turn are determined by wider economic, social, and political conditions (Liu et al., 2002). Socialeconomic inequalities are defined as “differences in the prevalence or incidence of health problems between individual people of higher or lower socioeconomic status” (World Health Organization, 2013). Commission on Social Determinants of Health of the World Health Organization (WHO) has singled out 10 determinants of health important for injustice in health: social gradient, stress, early life, social exclusion, employment, unemployment, social support, addiction, food, and transport (World Health Organization, 2008). Socialeconomic inequalities are usually measured by income, education, and occupation (Mackenbach et al., 2008; World Health Organization, 2010).

Strong associations between health and socioeconomic determinants have been documented in many studies (Mackenbach et al., 2008; Kaikkonen et al., 2009; Mackenbach, 2012). Considerable evidence suggests that lower socioeconomic status are associated with a poor self-perceived health, higher prevalence of chronic diseases (Radovanović et al., 2011; Lazic et al., 2012), and injuries, unhealthy behaviors such as smoking, inadequate diet, alcohol use (Jovanovic and Jakovljevic, 2011), and lack of physical exercise (De Looper and Lafortune, 2009; Dorjdagva et al., 2015). People of lower SES can expect to live less years in good health, have higher rates of mortality and die at younger ages (Mackenbach, 2012).

Socioeconomic inequalities are measured by various indicators of health such as life expectancy (Jakovljevic et al., 2015c), incidence of various diseases (Jakovljevic and Milovanovic, 2015), mortality, and self-assessment of health (Vuković et al., 2012). Self-assessment of health is one of the most commonly used health indicators recommended by WHO and European Union Commission (Janković et al., 2012). Self-assessed health is a commonly used measure of health status that asks individuals to rate their general health on five-point Likert scale with with five possible answers: very good; good; fair; bad; or very bad. The measure provides a valid and reliable assessment of overall health status, and has been found to be predictive of future health outcomes when used in national population health surveys (Park et al., 2015; Jakovljevic et al., 2016c). Also, it was found that the self-assessment of health is one of the important predictors of mortality, morbidity, functional limitations, and health care use in the population (Burström and Fredlund, 2001; Müters et al., 2005).

Comparative study of 22 European countries indicating that in almost all countries the prevalence of poorer self-assessments of health were significantly higher in groups of lower socioeconomic status (Mackenbach et al., 2008). People with lower level of education or income and unemployed persons are more likely to have poor self-assessed health (McFadden et al., 2008).

Despite the global wealth (Jakovljevic, 2016) and application of the best evidence-based interventions, socioeconomic inequalities in health are important, and ongoing public health problem in all European countries (Jakovljevic et al., 2016a) and a major challenge for the enactment and implementation of health policy (Jakovljevic, 2014). In Serbia, as in many countries in transition, socioeconomic inequalities in health have not been sufficiently studied, neither they receive due attention in public health policies (Jakovljevic et al., 2016b).

## The data report methods

### Public data set description—serbian 2013 national health survey

Data belonging to the 2013 National Health Survey for Serbia were observed (Results of the National Health Survey of the Republic of Serbia, 2013). These data were acquired using a cross-sectional studies on a representative probability sample of adult citizens aged 15 years or more (excluding Kosovo). The survey was conducted in accordance with the methodology and instruments of the European Health Interview Survey wave 2 (EHIS-wave 2). It was implemented by the Ministry of Health of the Republic of Serbia.

The sample consisted of all households listed by all enumeration areas of Census 2013. The mechanism used to generate a random sample of households and respondents is a combination of two sampling techniques: stratification and multi-stage sampling. Two-stage stratified sample of the population of Republic of Serbia was selected so as to provide a statistically reliable estimate of indicators that indicate the health of the population at the national level. As the main strata in the sample four geographic regions were identified: Vojvodina, Belgrade, Šumadija and Western Serbia, South-East Serbia. Their further division into urban and rural areas obtained a total of eight strata. The units of the first stage consisted of a total of 670 enumeration areas. The units of the second stage were households. Within each enumeration area 10 households were chosen (plus three backup households). Households were selected by means of linear sampling method of start and equal step of choice. In this way, households were selected with equal probability of selection and without repetition.

### Survey data description

Out of total 10,089 households contacted, 6500 of them agreed to participate in the survey, so that the response rate of households is 64.4%. Of the total of 16,474 registered household members aged 15 years and over 14,623 of them agreed to be interviewed, giving a response rate of 88.9%. Of this number of people who agreed to be interviewed, 13,756 of them accepted to fill in the questionnaire (response rate 94.1%). For the purposes of this study, the data on the adult population aged 15 years and over were used.

Data on demographic and socio-economic characteristics of the respondents and their own health assessment was obtained through a face-to-face interview carried out at home, while information at the level of the household was obtained by means of a household questionnaire. The questions were validated instruments based on the standard questionnaires from similar types of surveys were. Fieldwork was conducted in the period from 7 October to 30 December 2013, and for the purposes of the research 68 teams were formed. Each team consisted of two interviewers and one health worker. All respondents were informed about the purpose of the study and agreed to participate. Ethical Standards in Health study are in compliance with the international (World Medical Association Declaration of Helsinki) and the specific legislation of our country.

Of the independent variables, the researchers used demographic characteristics (age, gender, type of settlement, and marital status) and socioeconomic status (education, employment, and well-being index). Age of respondents was categorized into age groups of ten years: 20–29 years, 30–39 years etc. Gender is coded as male and female, place of residence as urban and rural, while the marital status was categorized as marriage or common law marriage and not married, divorced or widowed. Variables that reflect the socio-economic situation are education, which is designated as higher, secondary, and elementary, employment status as employed and unemplyed and household well-being index, according to which the population of Serbia, for the purposes of this paper, was classified into three socio-economic categories: rich class, middle class, and poor class.

A self-perceived health was used as the dependent variable and measured through a single question: “How do you regard your health in general?” Available responses were: very good, good, fair, poor, and very poor. For the present analysis, these responses were dichotomised into good (very good, good) and poor (fair, bad, very bad) health.

The data set has been submitted in a public repository Figshare and it is a available on: https://figshare.com/s/cfb6a5a0ef28427c0cdd. Data has been uploaded as Excel file while questionnaires are in PDF formats. Readers are free to access and reuse these publicly available data at the links provided above.

### Core socioeconomic inequalities in the country

There were more women (54%) than men (46%) in the sample. The highest percentage of respondents of both sexes belonged to the age group of 55–64 years. In the age group over 65 years there were more women. Slightly more than two-thirds of the respondents lived in a marriage or common-law marriage (69.3%). The highest percentage of respondents of both sexes has completed secondary education (60.8 and 48.6%), while there is the least of those who have higher education (17.4%). Among women there are significantly more of those who have completed elementary school or lower education (35.6%). In relation to the employment status the highest percentage belongs to the group of inactive population (59.4%). More than half of the respondents live in urban areas (55.3%). When it comes to well-being index, the largest percentage of respondents of both sexes belongs to the middle class (60.1%), followed by those who belong to the poor class (22.5%), and the rich class (17.4%). Distribution of health self-assessment of respondents by gender, age, marital status, type of settlement, education, employment status, and well-being index is shown in Table 1.

**Table 1:** **Distribution of individuals' self-assessed health according to demographic and socioeconomic characteristics**.

**Variables**		**Good health**		**Poor health**		***p***
		**N**	**%**	**N**	**%**	
**TOTAL NUMBER OF RESPONDENTS**						
Age	20–24	855	99.0	9	1.0	<0.0005
	25–34	1918	97.9	42	2.1	
	35–44	2080	95.2	106	4.8	
	45–54	2006	86.3	318	13.7	
	55–64	2215	76.9	664	23.1	
	65–74	1269	64.9	685	35.1	
	75–84	753	54.4	632	45.6	
	85+	101	50.5	99	49.5	
Gender	Female	5800	78.0	1632	22.0	<0.0005
	Male	5397	85.4	923	14.6	
Marital status	Has a partner	7446	82.7	1556	17.3	<0.0005
	Has no partner	3751	78.7	1013	21.3	
Employment status	Employment	4295	95.1	221	4.9	<0.0005
	Unemployment	6902	74.7	2334	25.5	
Place of residence	Urban	6549	84.5	1200	15.5	<0.0005
	Rural	4648	77.4	1355	22.6	
Education	Elementary education	2549	63.5	1463	35.5	<0.0005
	Secondary education	6544	87.9	901	12.1	
	Associated and Higher education	2104	91.7	191	8.3	
Well-being index	Rich class	2160	19.3	938	36.5	<0.0005
	Poor class	6839	61.1	1428	55.6	<0.0005
	Middle class	2198	19.6	202	7.9	<0.0005

Women more often evaluated their health as poor (22.0%) compared to men (14.6%). The age of respondent was inversely proportional to good health, the smallest percentage of respondents with poor health is among the youngest (1.0%), while there are most of those who rated their health as poor in the oldest age group over 85 years (49.5%). The difference of mean values of completed years between patients with poor and good health was statistically significant (*p* < 0.0005). The mean value of the number of years of the respondents with poor health was 65.58 ± 12.80 and with good 48.63 ± 16.86. Respondents living in marriage or common-law in a small percentage rated their health as poor (17.3%) as compared to those who do not have a partner (21.3%). Respondents living in rural areas were more likely to assess their health as poor compared to those who live in the city. The proportion of respondents with elementary education or less who assessed their health as poor (36.5%) is three times higher than in those with secondary education (12.1%) and four times higher than those with associated and higher education (8.3%). When it comes to employment status, the unemployed are five times more likely to assess their health as poor (25.5%) compared to employed (4.9%). A similar pattern was observed when it comes to the well-being index, i.e., Members of the poor class are three and a half times more likely to assess their health as poor (30.3%) compared to those who belong to the rich class (8.4%). Significant differences were observed between gender and all independent variables.

The results of bivariate and multivariate logistic regression for self-assessment of health as poor show that poor health is affected by age, gender, marital status, employment status, education, and well-being index (Table 2).

**Table 2:** **Odds ratios (OR) and 95% confidence intervals (CI) for poor self-assessed health depending on demographics and socioeconomic characteristics**.

**Variables**		**N**	**%**	**Binary logistic regression**		***P***
				**Univariate**	**Multivariate**	
Age				1.070 (1.066–1.073)	1.058 (1.053–1.062)	<0.0005
Gender	Male	6328	46.0	1.00	1.00	
	Female	7437	54.0	1.645 (1.505–1.798)	1.409 (1.256–1.580)	<0.0005
Marital status	Has a partner	4764	34.6	1.00		
	Has no partner	9001	65.4	0.771 (0.705–0.842)	1.014 (0.899–1.145)	
Employment status	Employment	9244	67.2	1.0	1.0	
	Unemployment	4521	32.8	0.152 (0.132–0.176)	0.557 (0.466–0.666)	<0.0005
Education	Associated and higher education	2296	29.1	1.0	1.0	
	Elementary education	4012	29.1	6.322 (5.380.7.4309	2.314 (1.856–2.887)	<0.0005
	Secondary education	7457	54.2	1.517 (1.288–1.786)	1.515 (1.234–1.859)	<0.0005
Well-being index	Rich class	2400	17.4	1.0	1.0	
	Poor class	3098	22.5	4.767 (4.047–5.616)	1.544 (1.223–1.949)	<0.0005
	Middle class	8267	60.1	2.279 (1.950–2.662)	1.311 (1.063–1.616)	0.011

With the aging, the number of respondents who assessed their health as poor increased. Odds ratio for the age is 1.058 (1.053–1.062), which means that each year more increases the risk of poor-health 5.8%. Women have 40.9% higher risk of poor health compared to men. Odds ratio for females is (*OR* = 1.409). Employees have about two times lower risk of poor-health in relation to the unemployed (*OR* = 0.557). The proportion of respondents who assessed their health as poor is inversely proportional to the level of education. Respondents with lower education are two and a half times more likely to assess their health as poor (*OR* = 2.314) compared to those with higher education. The same pattern was seen for the well-being index. Members of the poor and middle class more often evaluated their health as poor (*OR* = 1.544 and OR = 1.311) compared with those who belong to a rich layer of the population.

### Comparison with contemporary momentum elsewhere throughout europe

At the start of the 21st century, large differences in health still exist between and within all European countries, and some of these inequalities are widening (Mackenbach et al., 2007). Within EU countries, reported poor health among the population as a whole is most prevalent in Eastern European countries (Hungary, the Czech and Slovak Republics, Poland) (Mackenbach et al., 2008). Inequalities within countries between low and high income groups also exist in Western Europe (Iceland, Ireland, and the United Kingdom), but the proportion of persons reporting poorer health is low. The practice of unhealthy lifestyles, associated with a lack of information about health and healthy behavior, can contribute to poor health in Eastern Europe (Steptoe and Wardle, 2001). Differences in health status between East and West could be partly explained by differences in behavior related to health (smoking and alcohol consumption (Jakovljevic et al., 2013) and psychological factors (Laaksonen et al., 2001).

Substantial socioeconomic inequalities in self-assessed poor exist in Serbia (Jakovljevic et al., 2011). The results showed that there are significant differences in self-assessment of health, depending on the demographic and socio-economic variables (Jakovljevic et al., 2015b).

There is a positive correlation between age and health status of the population (Jakovljevic et al., 2015a). The older the respondents, the more they assess their health as poorer (Jakovljevic and Laaser, 2015). Women tended to report significantly worse health than men. For example, in the Ukraine the adjusted odds of women reporting their health as poor was 3.58 (Gilmore et al., 2002). Only in Estonia was no gender differential found in self-assessed health (Leinsalu, 2002). These findings are repeated in many studies that talk about the poorer health of women and the elderly (Szwarcwald et al., 2005; Espelt et al., 2008; Pappa et al., 2009). These findings could be explained by the fact that women have higher awareness of health issues and symptoms of the disease compared to men. The impact of place of residence on health varied by gender. In the Ukraine living in a village increased the risk of ill-health in women, but was not significantly associated with ill-health in men (Gilmore et al., 2002), whilst in Latvia rural men had a higher risk of ill-health, but place of residence was not a significant variable for women (Monden, 2002).

Education is one of the most important predictor of self-assessed health. In Baltic (Monden, 2005), as well as in many other European countries (Pikhart et al., 2001; Balabanova and McKee, 2002), people with a higher level of education in higher percentage assess their health as good. In Estonia, the risk of poor self-reported health amongst women with less than secondary education was 3.88 times higher than those with a university education, and for men the odds ratio was 2.32 (Leinsalu, 2002). These inequalities in health self-assessment could be explained by the fact that people with higher levels of education have more skills in coping with everyday life problems that could negatively affect their health as well as in overcoming them (Pappa et al., 2009). Poor and less educated people have fewer financial resources to solve their health problems, and despite higher morbidity and mortality, they less use health care services (Jakovljevic et al., 2014), and for some of them they have to pay proportionately more, compared to their income than the rich (Gwatkin et al., 2003).

Several studies reported that unemployed and economic inactive persons were more likely to report poor self-perceived health than employed persons (Monden, 2002; Molarius et al., 2007). Unemployed and their families have increased risk of poorer outcomes for health. Health effects begin already when people feel that their employment is uncertain. As the uncertainty grows, it increasingly acts as a chronic stressor whose effects increase with the length of exposure.

Reducing socio-economic inequality is one of the leading challenges for the adoption and implementation of health policies in many countries (Jakovljevic, 2013). The UN's Millennium Development Goals focused on poverty and development and reducing inequalities between countries (United Nations Millennium Development Goals, 2015). New European Policy for Health—“Health 2020” as one of the main objectives highlights improving health while reducing health inequalities (World Health Organization, 2013). The post-2015 era presents an opportunity for WHO and its partners to strengthen health inequality monitoring across all health topics at global, national and subnational levels. Improving health and reducing inequalities in health must be a common goal for all sectors of society (government and non-government sectors, organizations and institutions at national, regional, and local levels), which is the only feasible through joint integrated policies, strategies, and programs (Stahl et al., 2006).

## Conclusive remarks

The elderly, females, with a lower level of education, unemployed, and belonging to the lower socio-economic class, have poorer health. Socio-economic inequalities in health are a major challenge for health policy, not only because they represent social injustice but also because solving health problems of underprivileged groups of the population can influence the improvement of the health status of the population as a whole.

## Author contributions

All authors listed, have made substantial, direct and intellectual contribution to the work, and approved it for publication. SR and MJ developed research questions, designed the study, and prepared manuscript for this Data report. SK participated in the presentation and interpretation of the results, reviewing of the manuscript.

### Conflict of interest statement

The authors declare that the research was conducted in the absence of any commercial or financial relationships that could be construed as a potential conflict of interest.
